# Increased Levels of Serum IL-15 and TNF-β Indicate the Progression of Human Intracranial Aneurysm

**DOI:** 10.3389/fnagi.2022.903619

**Published:** 2022-06-17

**Authors:** Shuzhe Yang, Qingyuan Liu, Junhua Yang, Jun Wu, Shuo Wang

**Affiliations:** ^1^Department of Neurosurgery, Beijing Tiantan Hospital, Capital Medical University, Beijing, China; ^2^China National Clinical Research Center for Neurological Diseases, Beijing, China; ^3^Center of Stroke, Beijing Institute for Brain Disorders, Beijing, China; ^4^Beijing Key Laboratory of Translational Medicine for Cerebrovascular Diseases, Beijing, China

**Keywords:** unruptured intracranial aneurysm, serum cytokines, aneurysm progression, IL-15, TNF-β

## Abstract

**Objective:**

Existing evidence suggests that chronic inflammation promotes the progression of human intracranial aneurysm (IA) and many cytokines have been detected to participate in the process of inflammation. However, rare cytokines in plasma have been used as proxies for progression of IA. This study aimed to identify novel cytokines as biomarkers to predict the development of IA.

**Methods:**

Patients with unruptured intracranial aneurysms (UIAs) undergoing microsurgical clipping were prospectively recruited from January 2017 to June 2020 and were separated into two groups based on their ELAPSS score (low risk group < 10, intermediate-high risk group ≥ 10). Propensity score matching (PSM) was used to reduce imbalances in the baseline characteristics between groups. All blood samples were collected before surgery. A human serum 48-cytokines examination was performed to analyze the concentrations of serological cytokines. Clinical data and cytokines were compared between groups.

**Results:**

A total of 184 patients were enrolled in this study. The low risk group contained 77 patients and 107 patients were included in the intermediate-high risk group. Finally, there were 69 patients in each group after PSM with a matching rate of 1:1. The concentrations of 3 serum cytokines were significantly increased in intermediate-high risk patients, namely, interleukin-15 (IL-15), monocyte chemoattractant protein-1 (MCP-1), and tumor necrosis factor-β (TNF-β) (*P* < 0.05, |log_2_ fold change| > 2). The result of receiver operator characteristic (ROC)curve revealed that TNF-β had the highest predictive accuracy, with an area under the curve (AUC) value of 0.725 [95% confidence interval (CI) 0.639–0.811, *P* < 0.001] followed by IL-15 (AUC = 0.691, 95% CI 0.602–0.781, *P* < 0.001) and MCP-1 (AUC = 0.661, 95% CI 0.569–0.753, *P* = 0.001). Multivariate logistic analysis demonstrated high IL-15 [odds ratio (OR), 3.23; 95% CI, 1.47–7.12; *P* = 0.004] and high TNF-β (OR, 8.30; 95% CI, 3.25–21.25; *P* < 0.001) as the risk factors that correlated with intermediate-high risk of IA progression.

**Conclusion:**

UIA patients with intermediate-high growth risk exhibited increased serum levels of IL-15, MCP-1, and TNF-β. Serum IL-15, and TNF-β could serve as biomarkers to predict the progression of UIAs.

## Introduction

Intracranial aneurysm (IA) is a cerebrovascular disease characterized by a regional ballooning of intracranial arteries that occurs in approximately 3% of the population (Vlak et al., 2011). An increasing number of unruptured intracranial aneurysms (UIAs) are being detected with the common use of computed tomography angiography (CTA) and magnetic resonance angiography (MRA) (Brown and Broderick, 2014). IA rupture is the main reason for spontaneous subarachnoid hemorrhage (SAH) which leads to a high risk of death or disability. Existing studies have demonstrated that the growth of UIAs indicates aneurysm instability with an increased risk of rupture (Villablanca et al., 2013; Mehan et al., 2014). Thus, individual predictions of progression risk for UIAs is required to guide clinical decision-making.

The ELAPSS score has been developed to estimate the 3-year and 5-year growth risk of UIAs based on six patient and aneurysm characteristics and its effectiveness was verified by a large-scale study (Backes et al., 2017; Sánchez van Kammen et al., 2019). Accumulating evidence has revealed that inflammation plays a critical role in the pathogenesis of IA (Chalouhi et al., 2012a,b; Shimada et al., 2015). During inflammatory reactions, cytokines such as tumor necrosis factors (TNFs) and interleukins (ILs) can be produced. Accumulating evidence indicates that proinflammatory cytokines such as TNF-α, IL-6, and IL-1β participate in the formation or rupture of IAs (Morgan et al., 2006; Fontanella et al., 2007; Miyamoto et al., 2017). However, rare cytokines in plasma have been revealed as proxies for the progression of UIA. This study aimed to identify novel cytokines as biomarkers to predict the growth of UIA.

## Methods

### Study Population

This prospective study was approved by the Ethics Committee of Beijing Tiantan Hospital, Capital Medical University. Patients with UIAs undergoing microsurgical clipping were prospectively enrolled from January 2017 to June 2020. Written informed consent was obtained from all patients. The exclusion criteria of this study included: (1) patients with multiple UIAs; (2) patients with dissecting, fusiform, traumatic, and infectious UIAs; (3) patients with UIAs have treated prior to this admission; and (4) patients with other cerebrovascular diseases (cerebrovascular malformation, moyamoya disease, etc.). All enrolled patients were separated into a low risk group (0–9 points on the ELAPSS score) and an intermediate-high risk group (≥ 10 points on the ELAPSS score) based on a previous study (Sánchez van Kammen et al., 2019).

### Data Collection

Patient clinical data, including demographic information (age, sex, smoking history, and alcohol abuse history), medical history [hypertension, diabetes mellitus, hyperlipemia, previous transient ischemic attack (TIA), or ischemic stroke and coronary heart disease] and medications (aspirin and lipid-lowering medications), were obtained from electronic medical records. Aneurysm characteristics such as size, location and shape, were determined by CTA or digital subtraction angiography (DSA) by two experienced investigators (SZY and QYL, who have more than 5 years of experience reading neurovascular images). Based on the size of UIA, patients were divided into five groups and the origin of UIA was classified as the internal carotid artery (ICA), anterior cerebral artery (ACA), anterior communicating artery (ACOM), middle cerebral artery (MCA), posterior communicating artery (PCOM) and posterior circulating arteries (PCA, including the vertebral artery, basilar artery, cerebellar arteries, and posterior cerebral artery) according to the ELAPSS score. Regarding the shape of aneurysm, UIAs with a lobular or daughter sac were defined as having an irregular shape.

### ELAPSS Score

The ELAPSS score was developed to predict the progression of intracranial aneurysm (Backes et al., 2017). The ELAPSS score of each patient was calculated in this study. After we achieved the ELAPSS score, the estimated 3- and 5-year growth risk of an aneurysm was determined.

### Serum Cytokine Assays

Patient blood sample was acquired preoperatively to access serum cytokines. Blood samples were centrifuged for 10 mins at 15,000 rpm, and the supernatants were collected. All samples were stored at −80°C for further analysis. A total of 48 cytokines in human serum were measured by using the Bio-Plex Human Cytokines Screening Panel (Bio-Rad Corporation, Hercules, United States). Cytokine analyses were performed in accordance with the manufacturer’s instructions. Human serum 48-cytokines examination included following targets: CTACK, eotaxin, basic FGF, G-CSF, GM-CSF, GRO-α, HGF, IFN-α2, IFN-γ, IL-1α, IL-1β, IL-1ra, IL-2, IL-2Rα, IL-3, IL-4, IL-5, IL-6, IL-7, IL-8, IL-9, IL-10, IL-12 (P70), IL-12 (P40), IL-13, IL-15, IL-16, IL-17, IL-18, IP-10, LIF, MCP-1, MCP-3, M-CSF, MIF, MIG, MIP-1α, MIP-1β, β-NGF, PDGF-BB, RANTES, SCF, SCGF-β, SDF-1α, TNF-α, TNF-β, TRAIL, and VEGF. The serum samples were diluted with the standard diluent, then color-coded magnetic beads were diluted using the assay buffer and incubated samples for 30 min. After being incubated with the detection antibody, each sample was measured by Bio-Plex MAGPIX system (Bio-Rad corporation, Hercules, American).

### Statistical Analysis

Continuous variables are presented as the mean ± standard deviation (SD) or medians with ranges, and categorical variables are presented as number (%). Normality of distribution of continuous variables was assessed by the Kolmogorov-Smirnov or Shapiro–Wilk tests. Continuous variables and categorical variables were compared using Student’s *t* tests/Mann–Whitney *U* tests and Fisher’s exact tests, respectively. Receiver operator characteristic (ROC) curve analysis was performed to examine the predictive accuracy of serum cytokines with the value of the area under the curve (AUC). Each cytokine’s cut-off value was determined by calculating the maximum value of the Youden index (sensitivity + specificity − 1). With the cut-off value, the values of serum cytokines were defined as low (values below the cut-off value) or high (values above the cut-off value). A logistic regression model was applied to investigate the risk factors associated with aneurysm growth. Both univariate and multivariate analyses were performed, and variables with statistical significance in univariate analysis were incorporated into multivariate regression analysis which selected a backward model. The odds ratio (OR) and 95% confidence interval (CI) fofurther analysis. A totalr the OR are presented.

Propensity score matching (PSM) was used to reduce the imbalances in baseline characteristics between low risk and intermediate-high risk aneurysms. The propensity score of each patient was estimated by a logistic regression model. The following potential confounding factors were used as covariates: sex, medical history, and medications. Patients were matched with a match tolerance of 0.02 and a match ratio of 1:1. The nearest neighbor method without replacement was used. The expression of serum cytokines was compared between the two matched groups. Statistical significance was set as *P*-value < 0.05 with a two-tailed test. All statistical analyses were performed by using SPSS 26.0 (SPSS Inc., Chicago, IL, United States) and GraphPad Prism 8 (GraphPad Software, United States).

## Results

### Clinical Characteristics of Patients and Unruptured Intracranial Aneurysms

**FIGURE 1:**
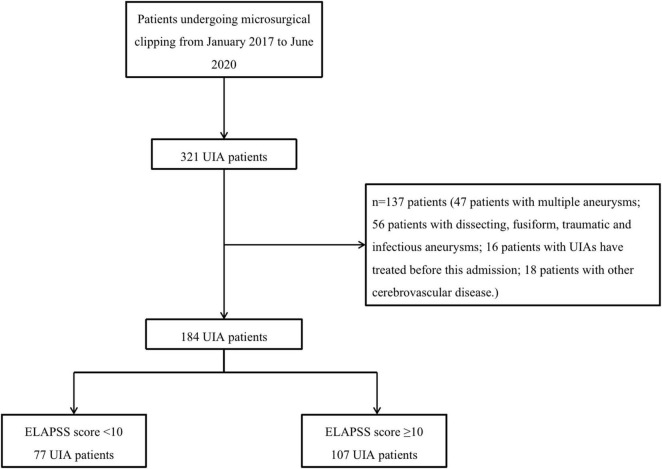
The flow chart of patient enrollment. UIA, unruptured intracranial aneurysm.

A total of 184 patients were enrolled in this study from January 2017 to June 2020. The patient inclusion flowchart is shown in Figure 1. Patient and UIA characteristics are listed in Table 1. Overall, 77 patients were included in the low risk group with their ELAPSS score less than 10 points and another 177 patients were enrolled in the intermediate-high risk group. Forty-nine (63.6%) patients in the low risk group were female, and there were 53 (49.5%) patients in intermediate-high risk group. Regarding their medical history, the number of patients who suffered from hypertension, diabetes mellitus and TIA or ischemic stroke was not significantly different between groups. However, more patients in the low risk group suffered from hyperlipemia and coronary heart disease than another group (*P* < 0.05). Regarding the characteristics of aneurysm, the size range of all low risk aneurysms was 3.0–4.9 mm, which differed from that of intermediate-high risk aneurysms (*P* < 0.05). In both groups, most aneurysms were in the ICA/ACA/ACOM (75.3 vs. 74.0%). Twenty-one (19.7%) intermediate-high risk aneurysms were irregular compared to low-risk aneurysms (3, 3.9%). The aneurysmal growth risk of both 3-year and 5-year in the intermediate-high risk group were significantly higher than that in the low risk group.

**TABLE 1:** The characteristics of patients and UIAs.

Characteristics	Before propensity score matching			After propensity score matching		
	Low risk	Intermediate – high risk	*P*-value	Low risk	Intermediate – high risk	*P*-value
No. of pts	77	107		69	69	
Age > 60 years, *n* (%)	26 (33.7)	34 (31.7)	0.777	22 (31.8)	22 (31.8)	1
Female, *n* (%)	49 (63.6)	53 (49.5)	0.058	43 (62.3)	41 (59.4)	0.728
**Medical history, *n* (%)**						
Hypertension	33 (42.8)	41 (38.3)	0.537	26 (37.6)	30 (43.4)	0.490
Diabetes mellitus	5 (6.4)	3 (2.8)	0.227	5 (7.2)	2 (2.8)	0.246
Hyperlipemia	15 (19.4)	7 (6.5)	0.008	7 (10.1)	7 (10.1)	1
Previous TIA or ischemic stroke	6 (7.7)	8 (7.4)	0.937	5 (7.2)	7 (10.1)	0.547
Coronary heart disease	9 (11.6)	4 (3.7)	0.038	3 (4.3)	3 (4.3)	1
Smoking	21 (27.2)	24 (22.4)	0.452	20 (28.9)	17 (24.6)	0.566
Drinking	6 (7.7)	7 (6.5)	0.745	6 (8.6)	5 (7.2)	0.754
**Medications, *n* (%)**						
Aspirin	5 (6.4)	8 (7.4)	0.798	2 (2.8)	6 (8.6)	0.147
Lipid lowering	12 (15.5)	6 (5.6)	0.025	5 (7.2)	5 (7.2)	1
**Aneurysm characteristics, *n* (%)**						
Size (mm)			< 0.05			< 0.05
1.0–2.9	0 (0)	0 (0)		0 (0)	0 (0)	
3.0–4.9	77 (100)	1 (0.9)		69 (100)	2 (2.9)	
5.0–6.9	0 (0)	29 (27.1)		0 (0)	17 (24.6)	
7.0–9.9	0 (0)	61 (57.0)		0 (0)	41 (59.4)	
≥ 10.0	0 (0)	16 (14.9)		0 (0)	9 (13.0)	
Location			0.725			0.484
ICA/ACA/ACOM	58 (75.3)	79 (74.0)		51 (73.9)	48 (69.5)	
MCA	18 (23.3)	23 (21.4)		17 (24.6)	17 (24.6)	
PCOM/posterior circulating	1 (1.2)	5 (4.6)		1 (1.4)	4 (5.7)	
Shape			0.002			0.005
Regular	74 (96.1)	86 (80.3)		67 (97.2)	57 (82.6)	
Irregular	3 (3.9)	21 (19.7)		2 (2.8)	12 (17.3)	
**Growth risk (%)**						
3-year	6.4 ± 1.4	17.0 ± 9.3	< 0.05	6.4 ± 1.4	16.8 ± 9.0	< 0.05
5-year	10.7 ± 2.3	26.8 ± 12.6	< 0.05	10.7 ± 2.3	26.4 ± 12.2	< 0.05

*UIA, unruptured intracranial aneurysm; TIA transient ischemic attack; ICA, internal carotid artery; ACA, anterior cerebral artery; ACOM, anterior communicating artery; MCA, middle cerebral artery; PCOM, posterior communicating artery.*

After PSM, 69 intermediate-high risk patients matched to 69 low risk patients. There were no significant differences in patients’ sex, age, medical history, and medication history between the 2 matched groups, however, the aneurysm size, aneurysm irregularity rate and aneurysm growth risk in the intermediate-high risk group were still significantly higher than those in the low-risk group.

### Serum Cytokines Between Low Risk and Intermediate-High Risk Aneurysms

**FIGURE 2:**
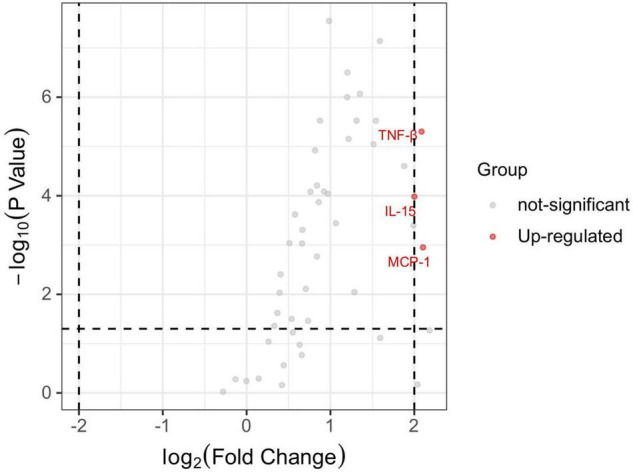
The volcano plot of differential expressed serum cytokines between low risk and intermediate-high risk patients. *P* < 0.05 and |log_2_ fold change| > 2 were used to identify the differential expressed serum cytokines. MCP-1, IL-15, and TNF-βwere significantly up-regulated in intermediate-high risk patients. IL-15, interleukin 15; MCP-1, monocyte chemoattractant protein-1; TNF-β, tumor necrosis factors-β.

**FIGURE 3:**
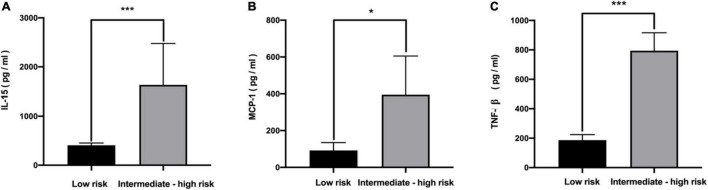
The concentration of serum IL-15 **(A)**, MCP-1 **(B)**, and TNF-β **(C)** between low risk and intermediate-high risk patients. *P < 0.05; ***P < 0.01. IL-15, interleukin 15; MCP-1, monocyte chemoattractant protein-1; TNF-β, tumor necrosis factors-β.

**FIGURE 4:**
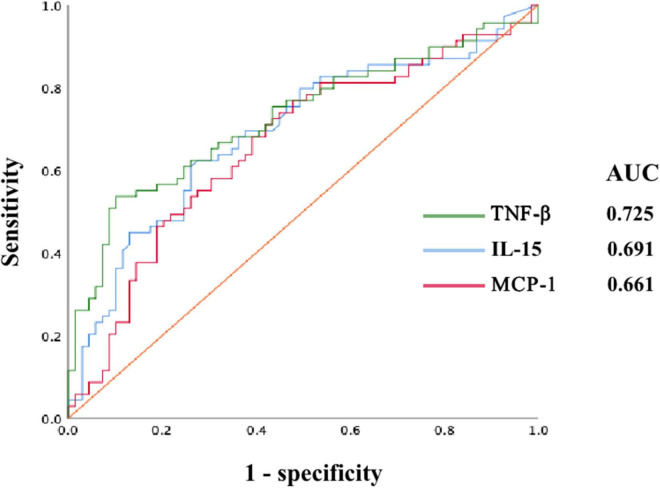
The receiver operator characteristic (ROC) curve of serum TNF-β, IL-15, and MCP-1. AUC, area under the curve; IL-15, interleukin 15; MCP-1, monocyte chemoattractant protein-1; TNF-β, tumor necrosis factors-β.

**FIGURE 5:**
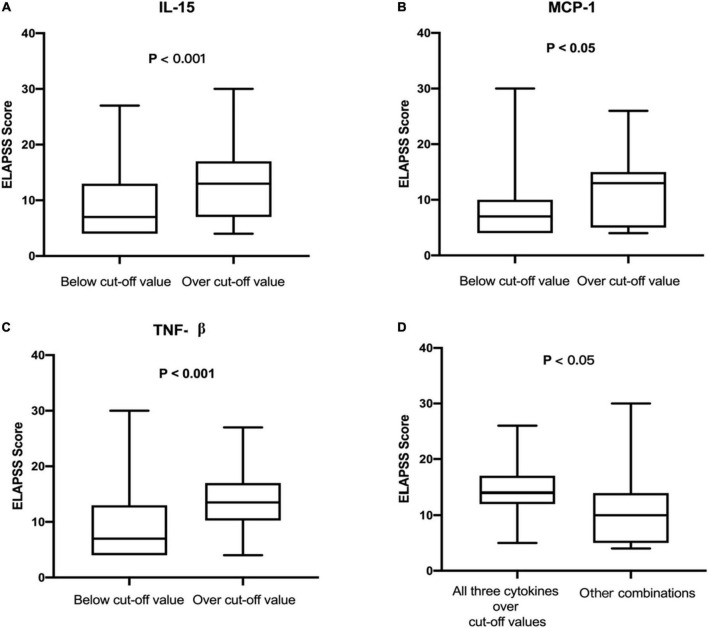
**(A)** The ELAPSS score between patients with the level of IL-15 below and over the cut-off value. **(B)** The ELAPSS score between patients with the level of MCP-1 below and over the cut-off value. **(C)** The ELAPSS score between patients with the level of TNF-β below and over the cut-off value. **(D)** The ELAPSS score of patients with high level of IL-15, MCP-1, and TNF-β was significantly higher than other combinations of these 3 cytokines. IL-15, interleukin 15; MCP-1, monocyte chemoattractant protein-1; TNF-β, tumor necrosis factors-β.

The levels of 48 serological cytokines were assessed between the low risk group and the intermediate-high group (Supplementary Table 1). Three cytokines, namely, interleukin-15 (IL-15), monocyte chemoattractant protein-1 (MCP-1), and tumor necrosis factors-β (TNF-β) were significantly increased in intermediate-high risk aneurysms compared with low risk aneurysms (*P* < 0.05, |log_2_ fold change| > 2) (Figure 2). The levels of these three cytokines in the low risk group and the intermediate-high group are shown in Figure 3. ROC curve analysis was applied to measure the predictive ability of these cytokines for aneurysm progression. The results demonstrated that TNF-β achieved the highest predictive accuracy, with an AUC value of 0.725 (95% CI 0.639–0.811, *P* < 0.001) followed by IL-15 (AUC = 0.691, 95% CI 0.602–0.781, *P* < 0.001) and MCP-1 (AUC = 0.661, 95% CI 0.569–0.753, *P* = 0.001) (Figure 4). Based on the ROC curve, each cytokine’s cut-off value was calculated. As shown in Table 2, higher risk of aneurysm growth was identified more frequently in patients with high levels of serum IL-15 (69.4 vs. 34.2%, *P* = 4.3 × 10^–5^, cut-off value = 576.07 pg/ml), MCP-1 (62.1 vs. 33.9%, *P* = 0.001, cut-off value = 31.65 pg/ml), and TNF-β (84.1 vs. 34.0%, *P* = 4.8 × 10^–8^, cut-off value = 309.59 pg/ml) than in patients with low levels of these cytokines. Furthermore, patients with high levels of all these 3 cytokines had a significantly higher growth risk of aneurysms than patients with other combinations of these 3 cytokines (88.0 vs. 56.0%, *P* = 0.004). Additionally, patients with levels of serum IL-15, MCP-1, and TNF-β higher than cut-off value had notably higher ELAPSS score, and the ELAPSS score of patients with high levels of all these 3 cytokines were significantly different from those of patients with other combinations of these 3 cytokines (Figures 5A–D). Univariate logistic analysis demonstrated that high levels of IL-15 (OR, 3.10; 95% CI, 1.40–6.87; *P* = 0.005) and TNF-β (OR, 7.16; 95% CI, 2.67–19.18; *P* < 0.001) were risk factors for aneurysm progression. Multivariate logistic analysis indicated that high IL-15 (OR, 3.23; 95% CI, 1.47–7.12; *P* = 0.004) and TNF-β (OR, 8.30; 95% CI, 3.25–21.25; *P* < 0.001) levels were associated with aneurysm progression (Table 3).

**TABLE 2:** The growth risk of UIA patients with serum cytokines over or below the cut-off value.

	Cut-off values (pg/ml)	Low risk	Intermediate-high risk	*P*-value
IL-15	< 576.07	50 (65.8)	26 (34.2)	4.3 × 10^–5^
	≥ 576.07	19 (30.6)	43 (69.4)	
MCP-1	< 31.65	39 (66.1)	20 (33.9)	0.001
	≥ 31.65	30 (37.9)	49 (62.1)	
TNF-β	< 309.59	62 (65.9)	32 (34.0)	4.8 × 10^–8^
	≥ 309.59	7 (15.9)	37 (84.1)	
Combination	All three over cut-off values	3 (12.0)	22 (88.0)	0.004
	Other combinations	33 (44.0)	42 (56.0)	

*IL-15, interleukin 15; MCP-1, monocyte chemoattractant protein-1; TNF-β, tumor necrosis factors-β.*

**TABLE 3:** The logistic analysis for the relationship between analyzed cytokines and growth risk of UIA.

Variables	Univariate		Multivariate	
	OR (95% CI)	*P*-value	OR (95% CI)	*P*-value
IL-15	3.10 (1.40–6.87)	0.005	3.23 (1.47–7.12)	0.004
TNF-β	7.16 (2.67-19.18)	< 0.001	8.30 (3.25–21.25)	< 0.001
MCP-1	1.47 (0.64–3.39)	0.36	–	–

*UIA, unruptured intracranial aneurysm; IL-15, interleukin 15; MCP-1, monocyte chemoattractant protein-1; TNF-β, tumor necrosis factors-β.*

## Discussion

This study sought to investigate the influence of serum cytokines on the progression of intracranial aneurysm. By comparing the levels of 48 serum cytokines between low aneurysm growth risk and matched intermediate-high aneurysm growth risk patients, serum MCP-1, IL-15, and TNF-β were found to be significantly up-regulated in intermediate-high risk patients. Furthermore, TNF-β achieved the highest predictive accuracy of aneurysm progression among the three cytokines. Finally, our analysis indicated that high levels of IL-15 and TNF-β were correlated with aneurysm growth, serum IL-15 and TNF-β may act as potential biomarkers to identify aneurysm growth.

Inflammation has been revealed as a critical player in the pathophysiology of intracranial aneurysms (Chalouhi et al., 2012a,b; Frösen et al., 2012; Shimada et al., 2015). During the formation of aneurysms, various kinds of inflammatory cells such as macrophages, accumulate in the aneurysm wall (Chyatte et al., 1999). Macrophages can release some proinflammatory cytokines, in turn, promoting the inflammation. Previous studies have revealed several cytokines that correlate with the formation or rupture of IAs (Morgan et al., 2006; Fontanella et al., 2007; Miyamoto et al., 2017). MCP-1 is a member of the CC chemokine family and is produced by monocytes, smooth muscle cells, fibroblasts, and vascular endothelial cells (Rollins et al., 1990; Colotta et al., 1992). In the early stage of aneurysm formation, endothelial cells are the main source of MCP-1 followed by smooth muscle cells; however, macrophages, endothelial cells and smooth muscle cells become the main sources of MCP-1 in the advanced stage of IA (Aoki et al., 2009). After secreted by endothelial cells, MCP-1 contributes to the proliferation and migration of vascular smooth muscle cells which leads to the formation and rupture of aneurysms (Chalouhi et al., 2012a,b; Ramji and Davies, 2015). Aoki et al. (2009) demonstrated that the expression of MCP-1 was up-regulated in human UIAs, which consistent with our study. In patients with ruptured IAs, the serum levels of MCP-1 were also up-regulated and could predict poor outcomes; in contrast, serum MCP-1 levels gradually decreased after these patients were treated with microsurgical clipping (Kim et al., 2008; Zhang et al., 2017). These findings suggest MCP-1 as a potential biomarker in either unruptured or ruptured intracranial aneurysms. MCP-1 is also involved in the pathophysiology of other vascular diseases. MCP-1 was highly expressed in human abdominal aortic aneurysm (AAA) samples as compared to normal aortas (Koch et al., 1993). In both human and experimental model mice, MCP-1 has been identified as a marker of atherosclerosis. Nelken et al. (1991) showed that MCP-1 was highly expressed in human atherosclerotic plaques through immunohistochemical staining. In experimental atherosclerosis, mice lacking MCP-1 suffered less lipid deposition and macrophage infiltration in aortic walls than wild-type mice (Gu et al., 1998).

IL-15 is a member of the IL-2 family that modulates inflammation, dictates T-cell response, regulates tissue repair and activates natural killer (NK) cells (Kong et al., 2014). IL-15 is widely expressed by various kinds of cells, including monocytes, macrophages, fibroblasts, epithelial cells and skeletal muscle cells. Existing evidence has revealed IL-15 as a cytokine that plays a central role in both the innate and adaptive immune response with the ability to activate NK cells, enhance the proliferation of memory CD8^+^ T lymphocytes and the cross-priming of CD4^+^ and CD8^+^ T cells (Chapdelaine et al., 2003; Picker et al., 2006; Huntington et al., 2009). Besides modulating immune response to protect the host from infectious agents, as a pro-inflammatory cytokine, IL-15 is also associated with some chronic inflammatory diseases. In vascular diseases, it has been demonstrated that IL-15 is up-regulated in both human and murine atherosclerotic lesions, moreover, interfering with the expression of IL-15 can reduce the size of atherosclerotic lesions in mice (Wuttge et al., 2001; Fisman and Tenenbaum, 2008). Furthermore, IL-15 is also involved in the pathophysiology of other cardiovascular diseases (CVDs) such as atrial fibrillation, myocardial infarction and myocarditis (Guo et al., 2020). Accumulating evidence indicates that inflammatory diseases of other systems, such as osteoarthritis, chronic pancreatitis, and inflammatory bowel disease, are also mediated by IL-15 (Kapoor et al., 2011; Tosiek et al., 2016; Manohar et al., 2018). However, whether IL-15 participates in the pathological process of IA is still unknown. IL-15 can induce inflammation through several methods, one of which is the regulation of certain inflammatory signaling pathways. Yan et al. indicated that IL-15 significantly regulated the inflammatory infiltration by affecting the nuclear factor κB (NF-κB) pathway. The same conclusion was also drawn by Gomez-Nicola et al. moreover, the IL-15 knockdown could inhibit the activation of the NF-κB pathway (Gomez-Nicola et al., 2010; Yan et al., 2016). As a classic inflammatory pathway, the key role of NF-κB in the formation and progression of intracranial aneurysms has been largely studied. Another method by which IL-15 promotes the inflammatory response is to release or induce the production of key inflammatory cytokines. It has been found that IL-15 can increase the production of IL-17, granulocyte macrophage colony stimulating factor (GM-CSF), interferon (IFN)γ, TNF-α, IL-8, and MCP-1 (Badolato et al., 1997; McInnes et al., 1997; Ziolkowska et al., 2000; Legroux and Arbour, 2015). The critical roles of TNF-α and MCP-1 in the formation and rupture of IA have been well investigated. Hoh et al. (2018) demonstrated that IL-17 was significantly expressed in both human and murine IAs; likewise, the inhibition of IL-17 could prevent the formation and rupture of IA. As a known regulator of monocyte and granulocyte function, GM-CSF has been shown to be positively correlated with IA size, but whether GM-CSF significantly expressed in IA patients compared with normal individuals needs further study (Chalouhi et al., 2015). In brief, as a pro-inflammatory cytokine, IL-15 can induce inflammation and then may promote the formation and rupture of IA through different methods.

The TNF superfamily of cytokines can regulate many physiological processes such as inflammation, cell differentiation, cell proliferation and cell death (Locksley et al., 2001). Numerous studies have revealed TNF-α as an important cytokine in inflammatory diseases, including IA, but studies of TNF-β in the pathophysiology of IA are scarce. TNF-β, also known as lymphotoxin-α (LTα), has a tertiary and quaternary structure similar to that of TNF-α, indicating similar biological activities of these two cytokines (Aggarwal et al., 1985). Etemadi et al. (2013) demonstrated that TNF-β could induce cell death, MAPK activation and NF-κB activation in the same way as TNF-α. Moreover, it has been reported that TNF-β is associated with some inflammatory vascular diseases. A previous study showed that TNF-βwas expressed in atherosclerotic lesions and that the loss of TNF-β significantly reduced the size of atherosclerotic lesions in mice (Schreyer et al., 2002). In addition, the expression of TNF-β in thoracic aortic aneurysm is significantly higher than that in normal aortic tissue and the TNF-β receptor (TNF-βR) is also up-regulated in AAA compared with control aortic tissue (Armstrong et al., 2002; Absi et al., 2003). Based on our results and existing studies, TNF-β may participate in the formation, progression or rupture of IA, which requires further research.

There are several limitations to this study. First, the concentration of cytokines in serum might be affected by comorbidities and the medication history of patients. Although we have adjusted the imbalances in recorded comorbidities and drugs through PSM, our results may still be influenced by this limitation. Second, as a prediction model for aneurysm growth, the accuracy of ELAPSS score has been confirmed (Feghali et al., 2021; Wang et al., 2021). However, this study lacked follow-up data, and we could not compare the real IA progression rate of our cohort with that estimated by the ELAPSS score, in other words, our results may not represent the real clinical situations. These limitations need to be addressed in our future work.

## Conclusion

This study compared the serum levels of cytokines between low and intermediate-high growth risk IA patients and was the first to indicate the correlation of serum cytokines and the progression of intracranial aneurysm. UIA patients with intermediate-high growth risk exhibited increased levels of serum IL-15, MCP-1, and TNF-β. Serum IL-15, and TNF-β could serve as biomarkers to predict the progression of UIAs.

## Data Availability Statement

The raw data supporting the conclusions of this article will be made available by the authors, without undue reservation.

## Ethics Statement

The studies involving human participants were reviewed and approved by the Ethics Committee of Beijing Tiantan Hospital, Capital Medical University. Written informed consent for participation was not required for this study in accordance with the national legislation and the institutional requirements.

## Author Contributions

SW was in charge of supervising the whole study. SY contributed to the conception or design of the work. SY and QL were responsible for drafting and revising. QL, JY, and JW were responsible for analysis and interpretation of data. All authors contributed to manuscript revision, read, and approved the submission.

## Conflict of Interest

The authors declare that the research was conducted in the absence of any commercial or financial relationships that could be construed as a potential conflict of interest. The reviewer YW declared a shared parent affiliation with the authors to the handling editor at the time of review.

## Publisher’s Note

All claims expressed in this article are solely those of the authors and do not necessarily represent those of their affiliated organizations, or those of the publisher, the editors and the reviewers. Any product that may be evaluated in this article, or claim that may be made by its manufacturer, is not guaranteed or endorsed by the publisher.
